# ‘Belonging’. ‘Patients' experiences of social relationships during pulmonary rehabilitation

**DOI:** 10.3109/09638280903464471

**Published:** 2010-02-16

**Authors:** Anne-Grethe Halding, Astrid Wahl, Kristin Heggdal

**Affiliations:** 1 Faculty of Health Science, Sogn og Fjordane University College, Førde, Norway; 2 Department of Medicine, Førde Hospital, Førde, Norway; 3 Faculty of Medicine, Institute of Nursing and Health Science, University of Oslo, Blindern, Oslo, Norway; 4 Department of Health, Oslo University College, Oslo, Norway

**Keywords:** Pulmonary disease, social support, qualitative

## Abstract

**Aim:**

To unpack and interpret descriptions of experiences of social relationships during pulmonary rehabilitation (PR) for people living with chronic obstructive pulmonary disease (COPD).

**Method:**

Inspired by interpretive phenomenology, individual qualitative interviews were conducted twice with 18 persons from COPD rehabilitation units in two general hospitals. Qualitative content analysis was performed.

**Results:**

Analysis of the interviews revealed the overarching theme of *belonging*. The participants emphasised social integration in rehabilitation groups as well as support from peers and health-care personnel as important dimensions of social relationships with regard to PR. Active participation in and engagement with the groups provided opportunities for patients to share their knowledge, encouraged mutual trust, and support and increased self-confidence, and motivation for self-care and further social participation. Integration in the groups and perceived support during PR made coping and adaptation easier and had a positive effect on quality of life.

**Conclusions:**

Patients' perspectives on PR were strongly influenced by certain facets of social relationships, such as social integration and social support. Patients', peers' and health-care professionals' strategies to promote social support and social integration should be further explored in the future, both in different contexts and for longer periods of time.

## Introduction

Chronic obstructive pulmonary disease (COPD) constitutes a major health problem and a leading cause of chronic morbidity worldwide. The natural course of the disease involves a progressive decline in lung function and exercise capacity, with a simultaneous increase in breathlessness, coughing, wheezing and sputum production [1]. In addition to these somatic problems, persons living with COPD describe feeling socially isolated and report suffering from negative emotions. Their personal integrity and self-esteem are threatened due to dependence on others and self-blame for the disability inflicted by their condition [2–5]. There is no complete cure available for COPD, although pharmacotherapy can decrease its symptoms and complications, especially in the disease's early stages [1]. Consequently, patients with the disease are confronted with extensive demands on coping resources during their illness.

Pulmonary rehabilitation (PR) is a recommended, though often unavailable, part of COPD management [1,6,7] that is intended to help people cope with the demands of this chronic illness. International guidelines [1,8] lay out the principal goals for PR as being symptom reduction, optimisation of functional status and increased participation in everyday activities to improve quality of life. The programmes vary widely, but usually include time-limited exercise training with nutritional counselling and education. They are often group-orientated. Although much effort has been made to evaluate PR programmes, COPD patients' experiences of the contextual and relational aspects of rehabilitation have rarely been explored. Some studies have explored the meaning of PR for people living with COPD. In these studies, patients have positively evaluated both peer support [5,9–12] and the support of professionals [5,11–14]. Patients describe their benefits from PR as strengthened hope, experience of control and self-confidence [5,10–13], as well as re-engagement and strengthened social participation [5,11]. Although patients' positive social relationships have been identified as just one of several components of PR, a deeper understanding of the meaning of social relationships in this context has, to our knowledge, not been described to date.

Social relationships are associated with health outcomes [15,16] and are therefore extremely important for rehabilitation. The social psychologist Cohen emphasised the three aspects of social relationships that are associated with health outcomes, namely social support, social integration and perceived quality of social relationships. He defined social support as ‘… a social network's provision of psychological and material recourses *intended to benefit an individual's ability to cope with stress*’ [15, p. 676], which involves instrumental, informational and emotional support. It is the perceived availability of support that promotes health by buffering the effects of stress in terms of psychological, behavioural and physiological responses. Cohen and coworkers [17, p. 54] further defined social integration as ‘participation in a broad range of social relationships’, which includes active engagement and a sense of commonality and identification with one's social roles. This social connectedness seems to benefit health directly by providing social control and information, with the potential to motivate people towards increased self-care and responsibility for others, as well as promoting a positive effect and self-worth [15]. These effects appear to be independent of stress. Thus, both social support and social integration have the potential to influence health and wellbeing. It is also important to note that social networks that involve negative interactions have the ability to increase stress [15,16,18].

There is an increasing awareness that rehabilitation should be patient-centred, designed and evaluated with subjects as partners and with their perspectives and societal contexts in mind [16,19,20]. Consequently, qualitative studies of patients' experiences of rehabilitation have the potential to further expand knowledge and lead to better practice [5,16,19,20]. We conducted a qualitative study, primarily designed to explore COPD patients' experiences of everyday life with COPD as well as experiences during the rehabilitation process. Our results revealed that social relationships in PR groups may be of great significance for patients in terms of coping and wellbeing. Our article aims to interpret and contextualise social relationship experiences in PR groups.

## Method

This study is part of a larger investigation designed to explore COPD patients' experiences of everyday life and rehabilitation. It employs a longitudinal descriptive design and was inspired by interpretive phenomenology. This life-world perspective includes the idea that the self is constituted through lived experience. Thus, human experience must be studied within its historical, societal and cultural contexts. Such a view takes into consideration the fact that individuals are self-interpreting and embodied, and that their being is constituted by temporality [21].

### Study setting and participants

We recruited a convenience sample [22] from patients enrolled in PR programmes in COPD rehabilitation units at two general hospitals. The selection criteria for participation in the PR courses were as follows: a verified COPD diagnosis from a lung specialist and the absence of medical or cognitive conditions that would prohibit participation. During the final PR session, the lead rehabilitation nurses informed the patients of our study. From 2003 to 2005, the nurses invited 33 persons to participate. Written information was given to those who were interested. The final sample consisted of 13 males and 5 females from 52 to 81 years of age. The sample showed variation concerning illness duration, described symptom intensity, activity and social network. The participants all lived in private homes; 12 lived with family members, and 6 lived alone. The occupational background of the participants included industrial workers, engineers, fishermen, transport workers, janitorial staff, health-care assistants and civil servants.

### The rehabilitation programme

The PR was organised as a 12-week (1 day per week) group-based outpatient course, conducted in a multidisciplinary fashion. Specialist nurses were in charge of day-to-day operations, lung specialists were responsible for diagnoses and treatment, and physiotherapists and occupational therapists were active members of the team. Baseline and end-point physical examinations, tests, subjective health status assessments and nurse consultations were performed. Assessments of individual needs, resources, challenges and goals were parts of the baseline nurse assessment and consultations. Lectures were held on COPD (causes, symptoms, treatment, medication and exacerbations), coping strategies, activity/relaxation strategies, smoking cessation, social rights and nutrition. Subjects concerning social support and social integration were not distinct parts of the lectures. However, the importance of participating in social activities and asking others for support were included in the nurses' general advices on coping. Patients were encouraged to bring family members to the PR sessions; however, this rarely happened. Group exercise sessions were arranged to help participants with everyday activities, through upper limb training and regular indoor and outdoor exercise. Adjustment of individual goals, strategies and plans for medication were the main components of the personalised treatment plan that was created for each patient at the end of the course. A sketch of the PR programme is provided in Table I.

**Table I:** A 12-week multidisciplinary outpatient PR programme: 1 day per week, conducted in groups of five to six patients.

Session 1		Sessions 2–11	Session 12
Medical consultation: physical examination, tests, subjective health status, treatment plan	30 min	Open group conversation led by nurse	Tests of subjective health status
	90 min	Supervised group exercise led by physiotherapist	Nurse/medical consultation: creation of individual treatment plan
Nurse consultation: assessment of individual needs and goals	60 min	Lunch break: the group eats together	
	60 min	Lecture	
	15 min	Break	
	45 min	Lecture	

### Data collection

Between 2003 and 2005, qualitative interviews were conducted twice with each participant within 2 months of the end of the PR course and again 1 year later to capture changes over time in participants' experiences of rehabilitation and function in everyday life. Two persons were not followed-up for a second interview because of death, and one participant could not be reached. The interviews focused the participants' experiences from everyday life with COPD prior to, during and after the recent PR course as perceived shortly after the PR and again 1 year later. The second interviews were more focused on the participants' main experiences; data were explored in-depth and allowed us to reach data saturation. Thematic interview guides were used for data collection (Table II). We recorded their spontaneous descriptions [23], and the participants were encouraged to bring up whatever they considered relevant for the study. Central topics were further explored. Each interview lasted 40–90 min; all interviews were performed in the participants' homes or at the researcher's office. With one exception, all interviews were recorded. The results presented in this article are limited to the participants' experiences from the PR-course, based on results from both interviews.

**Table II:** Thematic interview guides.

Thematic guide for interview 1
– Encouragement to speak freely about whatever he/she thinks is relevant for the study; his/her life before the illness, experiences from the onset of the illness, etc.
– Experiences from everyday life with COPD prior to PR; symptoms, problems, impact on everyday activities (self-care, functional performance, household activities, leisure activities, health care ….).
– Psychosocial changes associated with the illness (occupation, economy, family life, social participation, emotions, mood, …..)
– Descriptions of a good and a bad day
– Descriptions of ways to meet everyday illness' demands prior to PR.
– How the participant was informed about/recruited to PR.
– Rationale, expectations for his/her participation in PR.
– Experiences from the PR-period; evaluation of its contents, methods, organising, encounters with peers and health personnel, …
– Experienced benefit from PR (health, social benefit, change in self-care, activity, coping …)
– Descriptions of ways to meet everyday illness' demands after PR
– Suggestions for improvements of the program
– Additional comments/reflections

Thematic guide for interview 2
– Interviewer's reading and participant's comments on a transcript summary of the first interview
– How the participant perceives current everyday life. Events of significance to the participant the last year
– Reflections on experiences from PR and how participation in PR has influenced everyday life last year
– Descriptions of how he/she now meets everyday challenges, difficult incidents
– Descriptions of sources for support, relief and joy
– Experienced follow-up from healthcare last year
– Experiences from contact with peers the last year
– Additional comments/reflections

### Data analysis

We used qualitative content analysis with search for meanings [24]. Each interview was transcribed verbatim, thoroughly read and summarised to acquire a global understanding of each participant's experiences. The initial and follow-up interviews were analysed separately up to the final thematising. Meaning units were identified and condensed to preserve relevant core expressions. Using the N6software programme (QSR International Pty Ltd 2002), further coding, *categorisation*, and *thematising* analyses were performed. Common codes were created by comparing content across all interviews, ensuring that meanings remained coherent with the context of each interview. These common codes were clustered within content areas and further abstracted into categories and subcategories consistent with their joint meaning. For example, the meaning unit ‘We were able to share, it was no problem’ was coded as *sharing experiences* and categorised under *supporting phenomenon* in the subcategory *patient interplay*. In the final steps of the analysis, the subtheme *dialogue*, *shared understanding and fellowship in the groups* emerged within the overarching theme of *belonging*. Through all steps, including the interview, meanings were analysed, guided by the methodological perspective and the following research questions: What are the participants' concerns and experiences in the context of this PR programme? How do participants make sense of their rehabilitation experiences? How can patients' experiences within this PR programme inform rehabilitation practice?

### Trustworthiness

Throughout all data collection and analysis, *credibility, dependability and transferability* [24] were emphasised. During the interviews, we gave special attention to openness [25] by actively listening and by asking questions to elaborate on discussion topics. To explore the meaning of participants' responses, each code and category was interpreted in the context of the meaning of the broader transcript text. Two researchers worked on categorising and thematising via dialogue, challenging each other's preconceptions and interpretations of textual meaning. All of this was done to ensure *credibility*. To strengthen *dependability*, all participants were invited to comment on the transcribed summaries of their interviews. The themes from the initial and follow-up interviews were compared to search for changes and consistency. Characteristics of the participants and the context are presented here to facilitate the *transferability* of our results.

### Ethical considerations

The Regional Committee for Medical Research Ethics (REK nr. 211.03) and the Norwegian Social Science Data Services (nr. 10434) approved this project. Each participant returned a signed consent formula prior to telephonic contact from the interviewer.

## Results

The participants considered that the PR programme encouraged their sense of belonging because it provided fellowship and opportunities to share in the groups, as well as support from both peers and health-care professionals in an informal and cheerful atmosphere. Of note, participants also described barriers that prevented them from feeling a sentiment of belonging. They held the view that their participation in and the support provided by social relationships in the PR group made coping and adaptation easier and hence had a positive effect on their quality of life. The overarching theme, namely *belonging*, can be further elucidated under the following subthemes:

Belonging through cheerfulness and informal settings.

Belonging through dialogue, shared understanding and fellowship.

Challenges to belonging.

Belonging through professional care and competence.

The participants were grateful for the opportunity to attend PR sessions, and they contrasted their experiences with the perceived lack of previous support. They described how their everyday life had been challenging, without adequate strategies to meet the demands from their deteriorating health and uncertain futures. Falling ill with COPD had changed their life situations, and they felt quite helpless, lonely, often poorly understood by others, and to some degree neglected by health-care professionals and health-care systems.

### Belonging through cheerfulness and informal settings

The informal and cheerful atmosphere in the PR groups was a central condition for the participants' engagement and for the establishment of supportive social relationships, as it facilitated communication and made the gatherings pleasant. One of the male participants expressed his surprise at this:

‘Indeed we looked forward to Wednesdays! I work full-time but took sick leave these days. Wednesdays meant variation in my schedule, and I looked forward to attending. […] Under other circumstances a hospital is not a welcoming place. But those sessions were special. Obviously we are ill, but I kind of forgot it, because it was so nice to be there. The two other participants from my village agreed; it was just pleasant and cosy! I will hold onto this memory for a long time’.

The informal and cheerful tone in conversations with health-care workers and peers made the traditional patient and caregiver roles less prominent and facilitated equal participation and engagement. The sessions were more like pleasant, everyday social events than regular health-care encounters. The programme encouraged social participation, fellowship and wellbeing in spite of the patients' illness.

### Belonging through dialogue, shared understanding and fellowship

The rehabilitation groups provided opportunities to share experiences and to offer and receive support. For most participants, this was the first time they had met others who were living with COPD. This commonality was highly appreciated, and most of them felt readily included in their groups. In the groups they were able to compare their situations to peers', which was useful in helping participants to integrate their new identities as people living with COPD. The small groups enabled them to share their concerns and experiences by allowing sufficient time for ‘talking without interruptions’ and for asking questions:

‘We gained enormous trust and a sense of security from the start. Of course we had conversations about our illness, but we enjoyed small-talk as well. We were able to share; this was no problem, because we all understood one another. Sometimes these matters are just too difficult to talk about with people who don't have the disease’.

Time, trust and shared understanding facilitated group cohesion with dialogue about personal experiences. Opportunities to offer and to take advice from others were important. Several participants appreciated the resulting sense of mutual support: ‘We all contributed. We all used strategies to help us survive.’ The results suggest that participants shared their knowledge during PR and that this was an important part of their rehabilitation process. After finishing the course, several participants planned informally to continue their contact with other group members.

### Challenges to belonging

Some participants expressed indifference to the value of commonality and group support and claimed that ‘it meant nothing’. Feeling different because of one's differing needs and resources, being intimidated by group members who dominated the conversation, or being absent from several sessions were perceived challenges to group cohesion. These cases illustrate how some groups failed to encourage a sense of belonging and supportive social contexts. Analysis of these persons' experiences suggested that a perceived lack of commonality was connected to differences in personal background, personal interests and cognitive skills, or mistrust of the health-care system.

### Belonging through professional care and competence

The participants spoke positively about how health-care professionals in the PR programme served as caregivers, coaches and group leaders. Through caring competence, they provided support that relieved the patients' tension: ‘They seemed understanding and eager to help, and from the first moment we felt really welcome’! In particular, the nurses' initiative to write each of the patients an invitation was appreciated: ‘I found the rehabilitation really positive, most of all the fact that I received an invitation letter. That was very positive – it had never happened to me before’.

Reassuring and professional competence was demonstrated through physical examinations and tests, effective medication and skilled lectures and guidance. Through several years of illness, one of the participants had often longed for better provision of competent support: ‘It's not just about learning to live with COPD, learning to exercise and so on. In the PR course, I benefited from the medical treatment, supervision and advices of experts’. It was obvious that the PR programme offered easy access to specialists, which temporarily relieved some of their concerns about self-care and illness progression. Additionally, the nurses were attentive to individual health-related needs, experiences, concerns and opinions during group conversations. They took time to listen, gave personalised advice, and arranged for necessary additional medical assistance throughout the rehabilitation period. This attentiveness and care provided emotional and practical support in addition to knowledge about the disease and its treatment.

## Discussion

Our study reflects participants' sense of belonging in PR groups. The results were interpreted within the contexts of participants' everyday lives. The main theme was the need for a feeling of belonging and the desire for supportive social relationships, the latter supported by other studies of COPD patients who reported social isolation [2–5] and difficulties relating to others [26,27]. Participants experienced social integration and support as an important and positive part of PR that enhanced their quality of life. The PR groups represented new social networks that were of limited duration, providing opportunities for integration with engagement, commonality and role-identification, as well as resources to enhance their ability to cope with the stress of chronic illness. Our results are consistent with theories that emphasise the importance of social integration and perceived social support for health [15,16,18].

The majority of participants in this study experienced a high degree of social integration in contexts described as secure. Shared experiences and equality in terms of contributions and roles facilitated mutual trust, which is important to benefit from group support [15,16]. Another study of patients' experiences from PR described how patients preferred oral education in layman's terms that was conducted in groups, in addition to written material [9]. Such methods can encourage patient contributions and may promote a balanced dialogue among patients and professionals in a group context. Other studies have reported patients' benefit from peer support in rehabilitation groups [5,9–12]. However, our study also reveals how some participants seemed less socially integrated. Some of those appeared to be in need of special support, but these individuals were mistrustful of the health-care system because of previous negative experiences. These results indicate the importance of trust and social integration to perceived support in rehabilitation. Other cases appeared to involve less stressful life situations and, therefore, might have had less need for stress-buffering as derived from social support [15] within the PR groups.

The social context of the PR programme promoted the recognition and acknowledgement of personal experiences and led to new understanding, which is crucial in the reconstruction of self and in the active adjustment to changes imposed by chronic illness [16,18]. Heggdal's and Gullick and Stainton's studies have identified embodied knowledge and strategies for conscious body management as important factors that are often underestimated in coping with and recovery from a chronic illness [16,27]. In our study, the development and use of these resources were facilitated through participants' successful integration in PR groups.

The participants' opportunities to contribute positively to one another's wellbeing seemed to increase their positive self-esteem and motivate further self-care and contact with peers. In Western cultures, adults seem to value themselves by their ability to be independent, take care of themselves and to assist others [18]. The social networks of people who live with chronic illnesses often suffer from shrinkage, and mutual relationships can be threatened because significant others adopt a ‘giving role’ and over time become exhausted or disillusioned. The person who is living with the illness may withdraw from society to maintain control and independence [28,29]. However, persons living with COPD appreciate social participation and enjoy both receiving and contributing resources to society, in spite of their physical limitations [30,31]. Social integration and opportunities to nurture others have also been identified as predictors of health among patients with mental health problems [32]. Social connectedness and meaningful relationships have the potential to preserve personal integrity and increase motivation for self-care and responsibility for others. Physical health and functional status also seem to benefit from positive social relationships through a direct effect from social control and information [3,15].

Our study identified the health-care professionals as ‘significant others’ [18,33] who provided adequate support in the groups and in individual consultations. These results are consistent with the positive impact of social support on coping, wellbeing and health in chronic illness [15,16,18] and are also in line with the goals for PR [1]. Patients value open dialogue with health-care professionals who pay attention to their concerns and with whom they establish relationships characterised by trust, respect and power equality [18]. Patients' appreciation of support from health-care professionals during PR is supported by former studies [5,11–14]. It is important to note that it is the *perceived* availability of social support that has a positive impact on health [15,16,18]. In our study, emotional, informational and instrumental support [15] from health-care professionals and peers were closely connected and intertwined in the participants' experiences, thus seemingly reinforcing each other. Our participants underlined the importance of health-care professionals' knowledge, understanding and hope in a PR context, as earlier described by Heggdal in an analysis of patients' experiences across patient groups [16,28]. The life-threatening and anxiety-provoking aspects of COPD help us to understand the importance of available professional support. Trust in health-care providers has been shown to be an important element of social support in other COPD contexts [5,11,13,16,34]. Positive health-care encounters seem to influence patients' ability to seek support and to relate positively to their own functioning [28,35,36]. Continuity of support throughout the illness course is important to improve health status, to provide effective treatment for exacerbations and to delay COPD progression.

Traditionally, the patient is expected to be a recipient of health-care, and the ordinary doctor–patient relationship is often impersonal, technical and controlled by the doctor [18]. However, within the PR programmes, the atmosphere and norms were different, more informal, humorous, personal and less disease-oriented. This atmosphere contributed to the participants' wellbeing and their engagement in rehabilitation activities. Cohen's model of social relationships and health usually includes support provided by non-professionals [37]. Other authors do not make this distinction and include professional support in the broader notion of social support [16,18]. In the context of this study, a strict differentiation between social and professional support seems irrelevant. Professionals were perceived by their patients as integrated members of the groups. Their informal behaviour, participation and trust in patients' resources appeared to increase the participants' feeling of belonging and of being valued as individuals. These results are in line with a study from a PR context where family-like relationships were shown to be valuable [11]. In a study of the rehabilitation of drug abusers [33], trivial and informal ‘commonplace’ situations with staff and residents seemed to be of great importance for perceived emotional support and positive enhancement of the residents' identities. The informal atmosphere and equality in roles during the PR in our study bear similar traits in terms of this ‘commonness’ [33]. Individuals were encouraged to take on an active role, to feel integrated and to share and discover new possibilities for rehabilitation.

PR guidelines only partly include the political vision of changing rehabilitation towards an emphasis on patient-centeredness, by accepting individual needs and assisting each patient towards social integration with society [1,8]. Shifting to a patient-centred perspective means focusing on vulnerabilities and on the need for support, as well as on their abilities and social participation options in the journey towards improved wellbeing and health, in spite of their chronic illness. In our study, social context played an important role. Based on our results and existing theory, we suggest that positive feelings, social integration and support throughout the PR course may have helped in building participants' self-confidence, motivation for self-care and improved social participation and trust of the health-care system. All these outcomes have the potential to improve patients' good health and wellbeing in the long-term.

### Limitations of the study

Local cultural and geographical influences must be considered when assessing the transferability of our results. The study was conducted in a geographic area where networking between the patients was often especially difficult due to physical distances. Fifteen PR participants during our study-period refused to take part in the research. These may have been participants who perceived social participation and support within these contexts differently. Further research in different contexts should study patients' and health-care professionals' perspectives in terms of contextual and relational phenomena.

### Implications for practice

Group PR for patients with COPD is recommended. Based on our results, we suggest that successful integration and mutual support within the rehabilitation groups be explicitly stated as a goal of any rehabilitation programme. In planning group interactions, one should encourage patients to use their experiences, knowledge and mutual support frameworks to promote successful integration, coping and health strategies. An informal atmosphere is recommended. Each group member's perceived group cohesion and social support needs should be assessed. If necessary, individual support should be provided. Education and guidance on strategies for social integration and support as a part of active adaptation to a life with COPD should be part of PR and if possible should include patients' existing social networks [15]. Professionals' active participation in group activities seems to be important. The group leaders must demonstrate high competence in guidance, leaderships of groups and treatment of COPD to enhance the ability of the group to provide social integration and support. Programmes in the future should explicitly focus on the central theme of belonging and should include a certain level of follow-up.

## Conclusion

We conclude that social relationships, including social integration and social support, are important components of *patient-centred* rehabilitation for people living with COPD. Integration in rehabilitation groups and support from peers and health-care personnel is important for patients' self-confidence, coping, wellbeing and motivation for further social participation. Positive social relationships facilitate the use and development of patients' knowledge and may enhance their ability to provide mutual support, which is important for patient health and for keeping up with the demands of self-care. Patients', peers' and health-care professionals' strategies to promote social support and social integration should be further explored in the future.
